# Aripriprazole induced severe oculogyric dystonia treated with electroconvulsive therapy(ECT)

**DOI:** 10.1192/j.eurpsy.2023.2142

**Published:** 2023-07-19

**Authors:** K. Bhat Shah, T. S. Bhat, J. S. Pawar, N. Pande

**Affiliations:** 1 Ophthalmology, JMF’S ACPM Medical College Dhule; 2 Psychiatry, JMF’S ACPM Medical College; 3 Psychiatry, Shri Bhausaheb Hire Medical College, Dhule; 4 Psychiatry, Orange City Hospital, Nagpur, India

## Abstract

**Introduction:**

Aripiprazole is the third generation Antipsychotic, and Dopamine serotonin system stabiliser.It is partial agonist at D2 and 5 HT1 A and antagonist at 5 HT2.Most commonly seen adverse effects are Akathesia, fatugue,insomnia and headache the major advanatage is less propensity for extrapyramidal side effects and lmetabolic side eefects.

**Objectives:**

To report a case of Schizophrenia treated with Aripiprazole 15mg/day developiing occular gyric crisis which was tratment resistant.

**Methods:**

We administered Electroconvulsvive therapy, bidirectional brief pulse constant current 8 ECTS, under General anesthesia with medical fitness .

**Results:**

Patient Showed complete resolution of Dystonia after second ECTs and Showed improvment in Pyschosis Parameterd. Assessment using Naranjo Protocol made.

**Conclusions:**

Electroconvulsive therapy therapy is viable alternative to manage Dystonia when medical treatment fails

**Disclosure of Interest:**

None Declared

